# Aerobic-Exercise and resistance-training interventions have been among the least effective ways to improve executive functions of any method tried thus far

**DOI:** 10.1016/j.dcn.2018.05.001

**Published:** 2018-06-14

**Authors:** Adele Diamond, Daphne S. Ling

**Affiliations:** Department of Psychiatry, The University of British Columbia, Vancouver, BC, Canada

We appreciate that our colleagues, Hillman et al. (2018), would like to conclude that aerobic exercise improves executive functions (EFs). We, too, would like to conclude that. However, the facts thus far indicate that aerobic exercise interventions (with greater or lesser cognitive and motor skill demands), resistance training, and yoga have produced the weakest results for improving EFs of any method tried. We refer to that evidence briefly below and discuss how physical activity (in ways that researchers have largely ignored) may indeed help to improve EFs. All of this is discussed in far greater depth in Diamond and Ling (in press), which systematically reviews 179 studies reported across 193 papers.

We would like to mention three important caveats: First, “weakest” evidence does not mean “no” evidence; 44% of aerobic-exercise studies and 25% of resistance-training studies have found at least suggestive evidence of EF benefits. Thus, some studies have demonstrated EF benefits from these activities. Compare that, however, to 79% of Cogmed^®^ studies and 100% of studies of taekwondo, t’ai chi, Chinese mind-body practices, and Quadrato motor training (which can all be considered mindfulness practices involving movement) finding at least suggestive evidence of EF benefits (see Table 1 below). Second, our focus is exclusively on EF outcomes. We are not saying that physical activity has shown weak benefits across all domains; we are saying that physical activity interventions have thus far shown weak benefits *specifically* for EFs. Ours was never meant to be a review of the whole exercise-cognition literature nor a review of the physical fitness, health, or neural benefits of exercise. Third, we are not saying that physical activity does not benefit EFs. There are reasons to think it does. We are saying that interventions used to try to prove that have generally met with disappointing results.

**Table 1:** Summary of Results for EF Benefits across All Program and Intervention Types.

Studies that reported results only for far transfer measures, except those that examined the effects of physical activity or mindfulness (because for so many of these studies all EF measures are far transfer), are not included here.

The results reported here (except for studies of physical activity or mindfulness) pertain only to near transfer measures, thus results for reasoning or fluid intelligence, except for studies that targeted reasoning in their training, are not included in the calculations here.

Studies that did not specifically train reasoning and included only reasoning or fluid intelligence measures are not included here.

There were too few studies in any of the following categories to include them here, though they appear in Tables 2 and 3 and are discussed in the paper: interventions that combined aerobic exercise with other things, neurofeedback, commercial computerized cognitive training (other than Cogmed), theater, piano, photography, quilting, and Experience Corps.

^1^Suggestive = more EF improvement *or* better EF post-test performance than control group on ≥50% of measures.

^2^Clear = more EF improvement *and* better EF post-test performance than control group on ≥67% of measures. Whenever a study did not report post-test scores or change, that study is not included in this column.

^3^15 Cogmed studies are included in our review. One study did not include near transfer measures and so does not appear in Table 1.

^4^13 N-back training studies are included in our review. Three did not include near transfer measures.

^5^Six complex span training studies are included in our review. One study did not include near transfer measures and so does not appear in Table 1. Two were non-computerized and are included under “non-computerized training” in Table 1 rather than under complex span training.

^6^The calculations here do not include near transfer measures that are complex span tasks themselves. Were complex span outcome measures included, the percentage of studies showing suggestive or clear evidence would remain the same. The other two columns would be 44% (18) for improvement and 41% (17) for post-test. It is clear that complex span training improves complex span performance, even on untrained tasks.

^7^The calculations here do not include near transfer measures that are task-switching tasks themselves. Were task-switching outcome measures included, the percentages of studies showing suggestive or clear evidence would remain the same. The other two columns would be 56% (39) for improvement and 37% (30) for post-test. It is clear that task-switching training improves the ability to switch between tasks, even on untrained tasks.

^8^If the FITKids studies are counted as three separate, independent studies, then for enriched aerobic exercise the results would be 47% (19) for suggestive evidence, 13% (15) for clear evidence, 36% (73) for improvement, and 15% (39) for post-test.

^9^19 studies of Aerobic Exercise with Cognitive and/or Motor Skills Demands are included in our review. One study included only and so does not appear in Table 1.

^10^One Yoga study did not do pre-testing.

^11^Two School Program studies did not do pre-testing.

As scientists we need to set the record straight. We show below that almost all of the many criticisms leveled by Hillman et al. (2018) of the summary of our review presented in Diamond and Ling (2016) are wholly incorrect or at best misguided. It does not advance science to mischaracterize what we said. We acknowledge, however, that two of the criticisms leveled by Hillman et al. are well-taken; we apologize for those errors. Correcting those errors, though, does not change our conclusions.

## 1 The overwhelming preponderance of evidence is that resistance training and aerobic exercise interventions have thus far generally not been successful in improving EFs

Diamond and Ling (2016) was part of a special issue presenting invited addresses from the *Flux International Society for Integrative Developmental Cognitive Neuroscience Meeting* in 2014. Both that paper, and the invited address on which it was based, were explicitly a brief summary of the initial findings of the systematic review by Diamond and Ling (in press). Diamond and Ling (in press) is an especially comprehensive and extensive review of interventions, programs, and approaches that have tried to improve EFs: “Previous reviews have focused on the large literature on cognitive training approaches to improving EFs or the large literature on physical activity approaches to improving EFs, often concentrating only on studies in children or adults. This review looks at *all* the different methods that have been tried for improving EFs (including cognitive training and physical exercise, but also all the other approaches) and at all ages (not only children or only the elderly)” (Diamond and Ling, in press)

To locate studies for review, “we searched PubMed and PsycNET for all publications that had any keyword, or word in the title or abstract, from both of the following sets (Set 1: evaluate, evaluation, intervention, program, randomized control trial, train, or training; Set 2: attention (apart from Attention Deficit Hyperactivity Disorder [ADHD]), cognitive control, cognitive flexibility, EF, inhibition, inhibitory control, fluid intelligence, mental flexibility, reasoning, self-control, self-regulation, set shifting, task switching, or WM)” (Diamond and Ling, in press). Initially that search was limited to papers published by 2014. (That search did not pick up some important papers, such as the seminal one by Kramer et al. (1999), since none of our search terms was in its title, “Ageing, fitness and neurocognitive function,” and since it had no abstract or keyword list, where terms included in our search might have appeared.)

Publication of Diamond and Ling (in press) had been expected in early 2016. When that was delayed we used the time to (a) systematically investigate the references cited in papers that had met our search criteria for still more studies meeting our 11 inclusion criteria (hence Kramer et al. (1999) appears in Diamond & Ling (in press)) and (b) include studies published in 2015.

With permission from Oxford University Press, we reproduce here Table 1 from Diamond and Ling (in press), which summarizes the results across 12 different approaches for improving EFs. No matter which index one looks at for assessing efficacy in improving EFs – and four indices are presented in the table – resistance training and aerobic exercise with greater or lesser cognitive demands fall at or near the very bottom as least effective in improving EFs.

## 2 Addressing criticism that relevant literature was omitted in Diamond and Ling (2016). Part 1: neuroimaging findings and studies in rodents

Hillman et al. (2018) suggest that we erroneously reached the conclusion that there is a lack evidence of efficacy of aerobic exercise interventions for improving EFs in part because we “[misrepresented] the state of the science due to omitted literature.”

Hillman et al. (2018) were particularly distressed that neuroimaging findings and studies in rodents were not discussed in Diamond and Ling (2016). They wrote:

“The above [neuroimaging findings] and non-human animal findings are among the strongest evidence opposing Diamond and Ling’s (2016) perspective, and their failure to include these articles, which are among the most highly cited in the field, demonstrates not only a lack of consideration for the empirical evidence opposing their view and lack of fidelity in their literature review, but also considerable bias leading to misrepresentation of the existing state of the field” (Hillman et al., 2018).

Such studies do not provide evidence opposing our view, much less strong evidence in opposition. We omitted those studies and findings precisely because they are not directly relevant. None of the rodent studies looked at effects on EFs. Effects on EFs are what our review was about; studies on effects on other things were outside the purview of our paper. Effects on the brain are also different from effects on cognition or behavior and were outside the purview of our review. (That said, the purview of our review was far from narrow. We included a larger number of intervention studies looking at EF outcomes, and a far more diverse array of kinds of interventions, than anyone ever has before. However, our review was about EF outcomes and only EF outcomes.)

One might argue, “Isn’t an effect on the brain relevant to EFs, since EFs obviously depend on the brain?” It is true that EFs depend on prefrontal cortex (PFC) and other interrelated neural regions. From evidence that an intervention has an effect on PFC it would indeed be appropriate to *hypothesize* that that intervention *might* have an effect on EFs. It is *unjustified* to conclude, however, that an intervention improves EFs just because the intervention produced a change in PFC or other interrelated structures. *The improvement in EFs has to be empirically demonstrated*. Indeed, time and again studies of physical activity (e.g., Chaddock-Heyman et al., 2013) and cognitive training (e.g., Rueda et al., 2005) have found that an EF intervention produced a change in neural activity with no discernible improvement in EFs at all.

Just because something might seem logical (e.g., that a change in a brain region that helps subserve EFs should mean an improvement in EFs has occurred) does not necessarily mean it is correct. Thus far there is a dearth of evidence that (a) neural changes after physical exercise interventions have been reflected in EF improvements or that (b) resistance training interventions or aerobic exercise interventions (with greater or lesser cognitive or motor skill demands) improve EFs.

There are many reasons why one might find a change in the brain but not in EFs including: (a) not every change in brain activity is beneficial (e.g., Poldrack, 2015), (b) the brain change(s) might not have reached a critical threshold to cause an effect on EFs, or (c) the change in neural activity might not be related to the EF-demands of the behavioral task and/or might not have occurred in a brain region directly relevant to EFs. To illustrate the last point, in Hillman et al. (2014) the P3 changes reported from posterior electrodes *might* have detected changes having their origin in the intraparietal sulcus or posterior regions of the superior or inferior parietal lobule (relevant for EFs) *or* they might have had their origin in the more anterior region of parietal cortex (not directly relevant to EFs).

It has long been known that although a brain region is active during performance of a task, even if its activation pattern appears to be closely task-related, that brain region might not be involved in subserving performance of that task. The most famous early example of this involved hippocampal activity during classical eyeblink conditioning. Hippocampal neurons markedly increase firing during classical eyeblink conditioning and their changes in unit activity precede and accurately predict learning and improved performance on the task. Based on that, it was proclaimed that the hippocampus was the critical neural substrate for classical eyeblink conditioning (Berger and Thompson, 1978; Berger et al., 1980). The problem was that if you lesion or remove the hippocampus, classical eyeblink conditioning is unaffected and the conditioned eyeblink response is still acquired at the normal rate (Solomon and Moore, 1975). Clearly, the hippocampus is not needed at all for standard classical eyeblink conditioning. Similarly, parietal cortex activation increases during performance of an EF task, delayed response (Chafee and Goldman-Rakic, 1998), but removing parietal cortex does not affect delayed response performance (Diamond and Goldman-Rakic, 1989).

One possibility that would be interesting to explore (and no one has thus far) concerning intervention effects on the brain translating into effects on EFs, derives from the oft-repeated finding that changes in the brain can show up earlier than changes in cognition or behavior (e.g., Bookheimer, 2000; Beason-Held et al., 2013). Thus it would be interesting to follow participants, randomly assigned to an experimental condition and to one or more control conditions, for some years, looking at neural activity and EFs yearly. One might find a change in neural activity after the first year but improvement in EFs might not be seen until perhaps Year 3.

Hillman et al. (2018) repeat their error in taking brain changes as evidence of cognitive benefits in criticizing Diamond and Ling’s (2016) characterization of Krafft et al.’s (2014a) findings:

“Additional misrepresentation of the literature may be found in their description of the Krafft et al. (2014c) study, which Diamond and Ling (2016) cite to support their position. However, inspection of the results demonstrates greater change in brain activation in the neural network supporting inhibitory control for the aerobic exercise group compared to the attentional control group (Krafft et al., 2014a)….[Diamond and Ling, 2016] selectively report the results as the brain function outcomes… were not described.”

As we’ve mentioned, Diamond and Ling (2016) were examining effects on EFs, not brain activity. Krafft et al. (2014b) found neither more improvement in EFs nor better EF post-test performance from aerobic exercise: “There was no significant group by time interaction in any cognitive measure, indicating that the exercise intervention did not differentially affect cognition compared to the control condition” (Krafft et al., p. 6). Since Krafft et al. found no change in brain activation to be related to a change in any EF and no effect of condition on any EF, the brain activation findings did not merit mention in a review of EF changes.

Three of the Hillman et al. (2018) co-authors (Erickson, McAuley, and Kramer) have previously been criticized and corrected by Coen et al. (2011) for making the same error they have made here in conflating brain changes with cognitive ones or over-interpreting brain changes as indicating that cognitive improvements have occurred:

“Contrary to both the title and abstract [of Erickson et al., 2011], there is virtually no evidence in this article that exercise improved memory. After 1 y there were no differences [in memory] between the exercise and control groups. [Quoting Erickson et al.]: ‘Both groups showed improvements in memory, as demonstrated by significant increases in accuracy….Response times also became faster for both groups….[T]he aerobic exercise group did not improve performance above that achieved by the stretching control group, as demonstrated by a nonsignificant Time × Group interaction.’

[A]lthough the aerobic exercise group improved on the memory task, so did the stretching control group in whom hippocampal volume decreased, further undermining any assumed link between hippocampal volume and improved memory. However, just such a link was explicitly drawn in the abstract, which states ‘here we show, in a randomized controlled trial with 120 older adults, that aerobic exercise training increases the size of the anterior hippocampus, leading to improvements in spatial memory.’ Unfortunately both the title and abstract are misleading and a major overstatement of the findings.” (Coen et al., 2011, p. E89)

It is a basic and important principle that brain changes should not be over-interpreted as ipso facto indicating cognitive improvements.

## 3 Hillman et al. (2018) asserted that we do not understand the brain bases of motor function

Oddly, Hillman et al. (2018) felt the need to lecture us on the brain: “Neural circuits that support many aspects of motor function and motor learning including the cerebellum, basal ganglia, motor cortex, supplementary motor area, and cingulate cortex are intimately linked with brain circuits supporting executive function and other higher order cognitive functions (Caligiore et al., 2017; Lanciego et al., 2012; Strick et al., 2009).”

It is odd because Diamond wrote a seminal paper on exactly this back in 2000 that is widely cited, including by almost a dozen papers cited by Hillman et al. in their rebuttal, though ignored in the rebuttal itself. Back in 2000, Diamond wrote:

“[T]he cerebellum is a neuroanatomical structure important for movement that appears (1) to function in a circuit with prefrontal cortex, (2) to play a role in cognitive functions, and (3) to be affected in children with cognitive neurodevelopmental disorders.” (p. 49) “[A] similar argument could be made with reference to the caudate nucleus.” (p. 49) “[I]t makes sense that not only may the cerebellum and striatum play a role in cognition, but dorsolateral prefrontal cortex may contribute to motor performance. Dorsolateral prefrontal cortex has extensive interconnections with regions of frontal cortex more directly involved in motor functions such as premotor cortex and the supplementary motor area (SMA) (on premotor cortex, see Barbas and Pandya, 1987; Dum and Strick, 1991; Kunzle, 1978; on SMA, see Tanji, 1994; Wiesendanger, 1981)….Premotor cortex and the SMA in turn have strong interconnections with motor cortex, which is also a region within frontal cortex. Hence, dorsolateral prefrontal cortex is positioned to be in close communication not only with subcortical regions important for motor function but with cortical centers important for movement as well.” (p. 50)

Clearly, the statement by Hillman et al. that “the oversimplification of the human motor system, and brain networks supporting them, is at the very heart of the misguided nature of Diamond and Ling’s review” could not be further from the truth.

## 4 Addressing criticism that relevant literature was omitted. Part 2: reviews and research reports

Hillman et al. (2018) were distressed that several important and high quality reviews and empirical reports were not discussed in Diamond and Ling (2016). A closer examination reveals that criticism was misplaced. Hillman et al. were highly critical of us for not discussing the reviews by Vazou et al. (2016) and Donnelly et al. (2016). The first page of Diamond and Ling clearly states that our paper was submitted, accepted, and appeared online in 2015. These two reviews appeared the following year. Having said that, we will address the evidence presented in those papers (and others referenced by Hillman et al.) in this response. As we will show below, these newer papers do not counter or challenge the conclusions we stated in 2016.

Hillman et al. (2018) also took issue with our not including several empirical reports of cognitive outcomes from physical activity in children or adults. However, ***no*** study referred to by Hillman et al. that met our search and our inclusion criteria was omitted from our review. Most of the studies Hillman et al. took us to task for not including were solely correlational, included no control group, included no EF outcome measure, and/or looked only at acute effects immediately after a single, isolated exposure – all explicitly excluded from our review for reasons we took pains to explain.

Of note, Hillman et al. criticized us in particular for not including the Kramer et al. (1999) study. That is indeed an important study. We did not omit it for any nefarious reason, as Hillman et al. suggest, but simply because it met neither of our search criteria (described above). Note, however, that the Kramer et al. study *is* included in Diamond and Ling (in press) and as the reader can see in Table 1 above and Table 2 below, its inclusion does not change our conclusions. Kramer et al. found strong evidence for aerobic walking improving EFs, but only one other of the 17 studies of ‘plain’ aerobic exercise we reviewed found strong evidence of EF benefits. (Note, we restricted our review primarily to studies of healthy participants and those with ADHD, including only a random sample of 10% of studies of participants with medical conditions. Thus most studies of aerobic walking with adults with medical conditions were not included in our review.)

Hillman et al. (2018) criticized us for not describing our inclusion criteria well and for not defining our search terms or the search process. As explicitly stated in Diamond and Ling (2016), however, this was meant as a summary of a much longer paper (Diamond and Ling, in press). There was not room to include all the detail that appears in the longer paper. Thus we summarized our inclusion criteria at length on page 35 of Diamond and Ling (2016) in three long paragraphs spanning half a page, and left for the longer paper the fully itemized list along with the list of our search terms and description of the search process. It is the longer paper that is the systematic review; Diamond and Ling (2016) merely touched on some of the conclusions and referred readers for a more detailed and in-depth report to the longer paper.

## 5 What other reviews of the literature on the effect of ‘plain’ aerobic exercise interventions on EFs have concluded: are they in agreement, or at variance, with the conclusions of Diamond and Ling (2016)?

Hillman et al. (2018) argued that contrary to what Diamond and Ling (2016) concluded, there is stronger evidence of cognitive benefits from aerobic exercise with minimal cognitive demands (‘plain’ aerobic exercise) than from aerobic exercise with greater cognitive and/or motor skill demands. Actually the results seem equally disappointing for both. For all four dependent measures (Columns 1, 2, 3, and 4 in Table 1). ‘enriched’ aerobic exercise comes out slightly ahead. Virtually every recent review has come to the same conclusion about plain aerobic exercise as Diamond and Ling:

A Cochran Review meta-analysis of 12 randomized controlled trials (RCTs) in older, cognitively-healthy adults concluded that: “Overall none of our analyses showed a cognitive beneﬁt from aerobic exercise even when the intervention was shown to lead to improved cardiorespiratory ﬁtness….Our analyses comparing aerobic exercise to any active intervention showed no evidence of beneﬁt from aerobic exercise in any cognitive domain. This was also true of our analyses comparing aerobic exercise to no intervention” (Young et al., 2015: p.1).

In their review of 25 RCTs involving healthy older adults, Kelly et al. (2014, p. 28) concluded that “there is a lack of consistent evidence to show that aerobic interventions… result in improved performance on cognitive tasks for older adults without known cognitive impairment.” They report that when aerobic exercise was compared with stretching or toning, studies report more EF benefits from aerobic exercise on only two out of 40 separate EF measures (5%) than from stretching or toning.

Similarly, when aerobic exercise has been compared with ‘no exercise’ active control conditions, Kelly et al. (2014) report a similar lack of evidence showing more EF benefits from aerobic exercise; indeed, they report that on only two out of 38 EF measures (5%) have studies found more improvement from aerobic exercise than from no exercise active control conditions. Results were little better for aerobic exercise versus no treatment: Kelly et al. report that on only five out of 41 EF measures (12%) have studies found more improvement from aerobic exercise than from no treatment.

Gates et al. (2013, p. 1093) report that in their meta-analysis of 14 RCTs of aerobic exercise interventions involving older adults with mild cognitive impairment only “trivial, nonsignificant effects were found for executive function.”

Van Uffelen et al. (2008) reviewed five RCTs done with cognitively healthy older adults that looked at effects of aerobic exercise on EFs. Only one of those five studies (20%) found *any* benefit to EFs from aerobic exercise compared with control participants.

None of the above reviews were cited by Hillman et al. (2018). One review concluded that aerobic activity *does* improve EFs of older, sedentary adults (Colcombe and Kramer, 2003). That was co-authored by one of the co-authors of Hillman et al. (2018) and unlike all the other reviews above, it *is* discussed in Hillman et al. On the small number of studies reviewed by Colcombe and Kramer (2003) we do not disagree with their conclusions. It is simply that a great many studies have been published since 2003.

## 6 Addressing criticism that findings were misinterpreted or misrepresented by Diamond and Ling (2016). Part 1: Smith et al. (2010)

Hillman et al. (2018) felt strongly that Diamond and Ling (2016) misrepresented Smith et al.'s (2010) findings. Ironically, Hillman et al. misrepresented our representation of Smith et al.’s findings. Hillman et al. wrote that we said Smith et al. did “not find a relationship with executive function.” Yet, in the sentence immediately after that, to support their statement, Hillman et al. quote us as saying “little or no EF benefits from aerobic activity (Angevaren et al., 2008 [which included 11 studies]; Smith et al., 2010 [which included 17 studies]).” (Diamond and Ling, 2016, P. 37). We stand by that statement. Little benefit for EFs was reported by Smith et al.; no benefit to EFs was reported by Angevaren et al. Further, no benefit to working memory was reported by Smith et al. (and we, as well as most EF researchers, consider working memory to be a component of EFs; e.g., Araujo et al., 2017; Blair, 2017; Blakemore and Choudhury, 2006; Devine et al., 2016; Diamond, 2013; Gueron-Sela et al., 2018; Moriguchi et al., 2016; Ursache and Noble, 2016; White et al., 2017).

Diamond and Ling (2016) presented the figure showing effect sizes for EF benefits from Smith et al. so that readers could see the results for themselves. Of the 19 RCTs that looked at the effect of aerobic exercise on EFs in Smith et al.’s analyses, only one found an effect size significant at p < 0.05 and that effect was significant at p = 0.049. Two of the three studies showing the largest effects were not really studies of the benefits of aerobic exercise (hence in the quote above we referred to the number of RCTs looking at EFs in Smith et al. as being 17): Scherder et al. (2005) looked at slow, self-paced walking (which is not aerobic) and Masley et al. (2009) looked at the benefits of stress management plus a dietary intervention plus aerobic exercise (which does not permit conclusions about the benefits of aerobic exercise per se). It is unclear what Smith et al.’s mean effect size for aerobic exercise benefits to EFs would have been without those two studies, but certainly it would have been smaller and probably not significant. Of 12 RCTs looking at effects of aerobic exercise on working memory, Smith et al. report that none showed working memory benefits. If the studies Smith et al. included under working memory were combined with the studies they grouped under EFs, the mean effect size for that combined set of EF studies would not have been significant.

## 7 Addressing criticism that findings were misinterpreted or misrepresented by Diamond and Ling (2016). Part 2: studies from Hillman’s lab

Hillman et al. (2018) felt we misinterpreted the findings of two studies from Hillman’s lab (Hillman et al., 2014; Kamijo et al., 2011) and accused us of not understanding statistics. We fully stand by what we said however. In the Hillman et al. and Kamijo et al. studies, the physical-activity intervention group and wait-list controls did not differ at post-test on any EF measure, although on some EF measures the intervention group improved more than the control group. There is no disagreement between Hillman and ourselves about that statement of fact. The primary way that pair of findings can happen – differential improvement without differential final outcome – is if the two groups were not equal at the outset. The primary way one finds more improvement without better final post-test scores is for one group to start off better than the other, and either for the initially better-performing group to get worse (which raises eyebrows when the participants happen to be children, as in these two studies) or for the other group to catch up. One can see that both of those happened from the figures in Hillman et al. and Kamijo et al. reproduced in Diamond and Ling (2016). Such catch-up could easily arise from different developmental timetables and not from anything an intervention did. It is important to see both better improvement *and* better final performance to conclude that an intervention really improved the skill or ability in question.

Since Hillman et al. (2016) took issue with what we said about Kamijo et al.’s (2011) results for the one EF measure they report, the Sternberg test, we asked Saul Sternberg himself (inventor of the test) for his opinion. He agreed with our critique and went further to write,

“The performance of their subjects is astonishingly poor. (E.g., with three letters, the accuracy of the pretest performance is only 8% above chance!) There are studies with children – even retarded children – that report orderly and short reaction times and high accuracies: For example, see Harris and Fleer (1974), Cooney and Troyer (1994), Marroun et al. (2014), and White et al. (2011). Why is the accuracy so low, and the reaction times so long? One possibility is that the subjects weren't given suitable incentives, feedback, or practice. Another is that embedding the probe among other symbols impaired its discriminability. Also, the group difference at pre-test is huge! Given that it was a pretest, it isn't clear why the two groups couldn't have been approximately matched." (personal communication, Dec. 11, 2017; quoted verbatim with permission)

## 8 Aerobic activity with greater or more minimal cognitive demands

We acknowledge the legitimacy of Hillman et al.’s (2018) criticism that the choice of wording in Diamond and Ling (2016) – contrasting ‘mindless’ aerobic exercise with “exercise that includes cognitive challenges” (p. 40) – was unfortunate in that it set up too stark a dichotomy. It is a continuum, not either/or. In Diamond and Ling (in press) we used different terminology, referring to “aerobic exercise with minimal cognitive demands (‘plain’ aerobic exercise)” and “aerobic exercise enriched with cognitive and/or motor skill components (‘enriched’ aerobic exercise).”

Hillman et al. (2016) went on to write, “More importantly, however, is the use of the term ‘mindful’.” However, we never used the term ‘mindful’ to refer to physical activity that presents more cognitive challenges. We agree with Hillman et al. that athletes often operate in very cognitively demanding and complex competitive environments. Certainly athletes may need good EFs to perform optimally (e.g., Vestberg et al., 2012). We pointed out in Diamond and Ling (2016, p. 39), however, that while regular exercisers (athletes) might bring EFs to bear on activities such as running or jogging, novices randomly assigned to a running or jogging intervention might not. We elaborated on that point in Diamond and Ling (in press):

“For committed runners or joggers, for instance, these activities are ripe with cognitive challenges as they strategically plan how, or if, they want to trade off speed and distance, minimize extra steps, etc., or these activities can become exercises in mindfulness for them or provide the opportunity for exercising mindfulness. That is unlikely to be true for first-time exercisers assigned to an intervention. Thus, those who maintain a regular running regime by choice may do so more planfully or mindfully than those new to running (assigned to do it in some study).”

Thus, demonstrating that athletes might use EFs for their usual physical activities, does not necessarily mean that non-athletes newly assigned to that physical activity will necessarily bring EFs to bear on that activity. That needs to be empirically demonstrated.

We do not disagree with Hillman et al. (2016) when they say, “yet when queried about their thought process during performance, [athletes] cannot recall what they were thinking about during competition.” That refers primarily to metacognition (monitoring one’s thought processes) and recall memory rather than to EFs, so it is not particularly relevant to the matter at hand. Just because you cannot reconstruct your thinking processes does not mean you were not using EFs.

Additionally, just because there is brain activity does not necessarily mean you are using EFs. Hence Hillman et al.’s statement that “patterns of brain activation underlying ‘mindless’ physical activity have been identified from both fine and gross (e.g., walking) motor actions (e.g., Dum and Strick, 2002)” does not show that EFs, or any kind of thinking, was involved in those activities. The brain is active whenever you do anything and even when you are not doing anything; that activity cannot be used to infer a cognitive state.

It is also true, on the other hand, as Diamond has pointed out numerous times (e.g., Diamond, 2012), that not all activities one might label as cognitively challenging necessarily continue to require EFs in experts. Once one is really good at something, one generally uses PFC and EFs less (except when there is change or something unexpected happens):

“After something is no longer new, those who recruit PFC *least* usually perform best (Garavan et al., 2000; Jansma et al., 2001). Why? PFC is the evolutionarily newest region of the brain. Other brain regions have had hundreds of thousands more years of evolutionary time to perfect their functioning. Thus, I need PFC to learn a new dance step, but later if I try to think about what my feet are doing while dancing, I will not dance well.” (Diamond, 2012: 68–69).”

Thus, novices struggling to perfect a skill might need to concentrate harder and recruit EFs more than those already highly trained at the skill.

## 9 What reviews of the effect of ‘enriched’ aerobic exercise on EFs have found

Above we discussed reviews of plain aerobic exercise; now we turn to reviews that also looked at aerobic interventions with more cognitive and/or motor skill demands. Surprisingly, Hillman et al. (2018) neglect to mention the latest review (on which Hillman is a co-author, along with Diamond and 17 highly esteemed experts on the effects of physical activity on cognition and academic performance across the globe). This systematic review of physical activity intervention studies in children concluded that “there is currently inconclusive evidence for beneficial effects of PA interventions on cognitive and overall academic performance” (Singh et al., 2018).

Hillman et al. (2018) do cite another review, however, also co-authored by Hillman, that came to a more sanguine conclusion: The review by Donnelly et al. (2016) concluded, “On the basis of the evidence available…PA has a positive influence on cognition” (p. 1197). This systematic review of cognitive benefits of enriched aerobic exercise studies in children included many types of studies (cross-sectional, longitudinal, cohort, and acute-effects) explicitly excluded from Diamond and Ling’s review because they do not permit one to draw causal inferences. Donnelly et al. did include 10 published papers reporting results from RCTs, which do permit causal inferences to be drawn, however.

They counted these as 10 RCTs, though there were actually only four in total: The two papers by Davis et al. (2007, 2011) were of the same RCT (the first paper contained a subset of the participants in the second). The three papers by Krafft et al., 2014a, Krafft et al., 2014b, Krafft et al., 2014c were of the same RCT (with the first two papers reporting on a subset of participants included in the third paper). The four papers on FITKids were of the same implementation of that program (Chaddock-Heyman et al., 2013; Kamijo et al., 2011; and Monti et al., 2012 included subsets of the participants included in Hillman et al., 2014).^1^ The tenth paper (Chang et al., 2014) compared more- to less-intensive soccer practice. It is unclear why Donnelly et al. omitted Fisher et al. (2011), Pesce et al. (2013), and Schmidt et al. (2015).

Chaddock-Heyman et al. found a benefit to speed of processing but none to EFs. Donnelly et al. noted that on a measure of inhibitory control (an EF component), Chaddock-Heyman et al. found significant improvements for those in FITKids but not for wait-list controls, but Donnelly et al. neglected to mention that when Chaddock-Heyman et al. directly compared the change scores for both groups they were not significantly different. Monti et al. (2012) did not examine any EF outcome. Krafft et al. (2014a) included no post-test measure of cognition. Chang et al. (2014) found no difference in cognitive outcomes between their two groups; both conditions might have benefitted EFs, or neither, it is impossible to know. To borrow a phrase from Coen et al. (2011), we find the conclusion reached by Donnelly et al. that “overall, the results of studies using RCT designs have consistently demonstrated significant improvements in the treatment groups, particularly for EF tasks” (p. 1204) to be “misleading and a major overstatement of the findings.”

The review by Vazou et al. (2016) referenced by Hillman et al. (2018) included many studies that looked at other aspects of cognition, not EFs. Since studies with and without EF outcome measures were combined in their analyses, as well as EF and non-EF outcomes within a study, it is not possible to draw any conclusion about possible benefits of physical activity specifically for EFs from this review.

Similarly, the other recent meta-analysis referenced by Hillman et al. (2018), that by Northey et al. (2017), does not report statistical analyses for EFs but only for ‘global cognition’ (which included attention and memory in addition to EFs), so it is not possible to draw conclusions about possible benefits of physical activity specifically for EFs from this review. (Northey et al. discuss the Gates et al. (2013), Kelly et al. (2014), and Young et al. (2015) reviews referenced above, making it all the more puzzling that those reviews were omitted from Hillman et al. (2018) since Hillman et al. discuss Northey et al., 2017 at length.)

## 10 Responding to Criticism that we Failed to Create a Balanced Perspective

Hillman et al. (2018) level a serious accusation against us, that we intentionally distorted the facts to support our point of view, reprimanding us that as scientists we “should not selectively identify data that supports [sic] our own perspectives.”

As mentioned above, we did omit an important study that found strong evidence of EF benefits from plain aerobic exercise (Kramer et al., 1999) but that was only because it had not met either of our search criteria so we had not found it.

Hillman et al. (2018) criticized us for not citing “Pesce et al. (e.g., Pesce, 2012; Pesce et al., 2013, 2016; Vazou et al., 2016).” However, we *did* cite Pesce (2012) and Pesce et al. (2013). The other two papers were not published until the year after our paper appeared online. The other studies Hillman et al. criticized us for not including did not meet our inclusion criteria.

This hardly shows a pattern of intentionally biasing the studies we reported to support our point of view. We included all relevant reviews we found. Surprisingly, in critiquing us, Hillman et al. ignored several of those.

## 11 Responding to criticism that we put forward unsupported ‘beliefs’

Hillman et al. (2018) criticized us for putting forward non-evidence-based assertions or what they derisively called “beliefs” based on some agenda we supposedly had, instead of conclusions based on the evidence at hand or offering testable hypotheses. That criticism, however, is unsupported and misguided. We put forward testable hypotheses derived from solid evidence and any conclusions we stated were also evidence-based. We had, and have, no axe to grind. We were not then, and are not now, wedded to any particular perspective, hypothesis, or conclusion concerning the efficacy of any approach for improving EFs.

The conclusion we came to then (that there was stronger evidence for aerobic exercise with more cognitive and/or motor skill challenges improving EFs than for aerobic exercise with little or no cognitive and/or motor skill demands) is the same conclusion many others have reached, including Best (2010), Ericsson (2017), Ericsson and Karlsson (2014), Moreau and Conway, 2013, Moreau and Conway, 2014, Moreau et al. (2015), Pesce (2012), Pesce and Ben-Soussan (2016), Sibley and Etnier (2003), Tomporowski et al. (2008), Tomporowski et al. (2011), Tomporowski et al.2015, and Vazou et al. (2016).

We offered several testable hypotheses based on empirical evidence of what impacts EFs, such as that some of the benefits of aerobic exercise might be mediated through improved sleep and/or improved mood, and that the approaches most successful at improving EFs might be those that not only directly train and challenge EFs but also indirectly support EFs by working to reduce things that impair them (such as poor health, loneliness, sadness or stress) and enhance things that support them (such as providing joy, building self-confidence, and engendering feelings of belonging to a group with an important shared goal). There is not room here, but evidence that sleep (or lack thereof), mood (positive or negative), stress, and social support (or its lack) impact EFs was provided in Diamond and Ling (2016) and is elaborated in Diamond and Ling (in press) and Ling, Kelly and Diamond (2016). We also encouraged the field to test Moreau and Conway’s (2014) hypothesis that programs characterized by complexity, novelty, and diversity (variety) would be the most successful at improving EFs.

When delay of the publication of the volume in which Diamond and Ling (in press) will appear provided us the opportunity to include yet more studies in our review, we reported that although we had predicted that aerobic activity with greater EF demands would improve EFs more than aerobic activity with minimal such demands, “*our prediction has not been confirmed*….In general, the results for enriched aerobic exercise are fairly comparable to those for plain aerobic exercise (see Table 1)” (Diamond and Ling (in press); emphases in the original; Table 1 from that paper is reproduced here as Table 1).

Indeed, we have a long track record of being guided by the evidence and of being more than willing to admit when we have been wrong or a hypothesis we offered has been disconfirmed. As the final co-author of Hillman et al. (2018) – Kramer – knows, Diamond has very publicly admitted that a hypothesis of hers, on which a study they collaborated on was based, was thoroughly wrong (Diamond et al., 2007). When Simpson and Riggs proposed a competing interpretation for findings of Diamond’s, Diamond invited Simpson and Riggs to collaborate with her on research putting their competing hypotheses to the test. In their joint paper reporting the results of their collaboration, Diamond wrote that the results clearly supported the interpretation Simpson and Riggs had offered, not her own (Simpson et al., 2012).

## 12 There is reason to suspect that physical activity may benefit EFs in ways the research literature has not yet captured, however

As we noted in Diamond and Ling (2016, p. 38): “People who are more physically active and have better aerobic fitness have better EFs than those who are more sedentary (*children*: Hillman et al., 2005; Scudder et al., 2014; Sibley and Etnier, 2003; *older adults*: Boucard et al., 2012; Colcombe and Kramer, 2003; Voelcker-Rehage et al., 2011; *all ages*: Etnier et al., 2006; Prakash et al., 2015).” That suggests to us that there may be EF benefits from physical activity that physical-activity-intervention studies have not been capturing.

Hillman et al. (2018) call for research to vary factors such as dose, duration, motivational status, modality, and intensity of physical activity to try to determine when and how physical activity improves EFs, echoing what was said in Diamond and Ling (2016).^2^ We have since come to think, however, that that approach unlikely to do much to advance the field. Variables such as dose and duration have been varied somewhat across studies and they explain little of the variance. Again, with permission of Oxford University Press we reproduce one final summary table from Diamond and Ling (in press) that compares aerobic exercise interventions (as of 2015) that were more or less successful in improving EFs on several study characteristics. As you can see in Table 2, there is no evidence of greater EF benefits from aerobic exercise programs that extend over more weeks or had longer sessions, and that is true whether the programs included more or fewer cognitive and/or motor skill challenges. That is counter to the conclusion of Colcombe and Kramer (2003), who concluded that longer duration aerobic-exercise interventions produced more cognitive benefits for older adults than shorter ones. Their conclusion was correct for the evidence on hand back then, but with additional evidence since then, it is no longer correct.

Neither is there evidence that studies that found more evidence of EF benefits had more power; there was no systematic difference in EF outcome measures and the mean number of participants per condition for studies finding greater EF benefits was smaller than in studies finding fewer executive functions benefits. Similarly, there is little difference in the mean age of older adults in studies finding more evidence of EF benefits and studies finding less.

Choice of control group does not seem determinative either; the percentage of measures on which a greater EF improvement was found from plain aerobic exercise than in control subjects was roughly 22% regardless of whether the control condition was standard PE, stretching and toning, or no treatment. A slightly larger percentage of the studies with older adults that found at least a suggestion of EF benefits included brisk walking as at least one component of their aerobic exercise program (100%) than studies with older adults finding little or no EF benefit (88%) included brisk walking as at least one component). A marginal advantage for brisk walking can also be gleaned from the fact that of those studies that found an EF benefit on over half of their measures, 66% used fast walking as their sole aerobic activity, whereas of those studies that found an EF benefit on less than a third of their measures, 38% used fast walking as their sole aerobic activity.

We suggested as a working hypothesis that perhaps aerobic exercise interventions have not been going about it in the right way. Until very recently, there have been no (zero) RCTs or quasi-experimental studies looking at the benefits of participating in a sport for improving EFs. Perhaps improving EFs is less about improving aerobic capacity per se or improving a particular motor skill, and more about touching hearts and minds. EF benefits from ‘enriched’ aerobic exercise interventions with more cognitive and motor skill demands have been no better than from ‘plain’ aerobic exercise (such as running on a treadmill), but the enriched programs have generally tacked on skills from sports (e.g., dribbling a basketball) in a decontextualized way, outside the context of actually playing the sport.

“It may be that the people need to engage in a sport, rather than do exercises drawn from that sport done out of context….Participants are more likely to be emotionally invested in a sport than in decontextualized exercises, and their emotional investment may be key to whether that activity, even if it challenges EFs, ends up improving EFs” (Diamond and Ling, in press). Recent studies are finding some preliminary evidence consistent with this hypothesis (e.g., Alesi et al. (2016) with soccer and Ishihara et al. (2017) with tennis). Studies of other real world activities (such as cooking, managing a budget, theater and Experience Corps) have also found evidence of benefits to EFs (e.g., Carlson et al., 2009; Noice et al., 2004; Wang et al., 2011; Willis et al., 2006).

“We predict that the activities that will most successfully improve EFs will include each of the following elements: (1) tax EFs, continually challenging them in new and different ways, (2) be personally meaningful and relevant, inspiring a deep commitment and emotional investment on the part of participants to the activity and to one another, (3) have a mentor or guide who firmly believes in the efficacy of the activity and sincerely cares about and believes steadfastly in the individual participants, and (4) provide joy, reduce feelings of stress, and inspire self-confidence and pride” (Diamond and Ling, in press).

Real world activities (such as sports) train diverse EF skills under diverse situations. Exactly the same situation rarely occurs twice in real life.^3^ It has long been known that varied practice (presenting novel situations for practicing a skill) leads to better long-term outcomes than constant practice (Ahissar and Hochstein, 2004; Bransford et al., 1979; Rosenbaum et al., 2001; Schmidt and Bjork, 1992; Shapiro and Schmidt, 1982). The physical-activity interventions that have been studied, however, often involved a fair bit of repetition and a limited set of contexts. They have also often focused on training individual skills one at a time. However in the real world, multiple skills are often required at once or in close succession.

**Table 2:** Duration, Dose, and Frequency for Aerobic Exercise Interventions broken down by Plain vs. Enriched Aerobics and by Whether EF Benefits were Found '"Plain" Exercise (e.g., brisk walking).

“Plain” Exercise (e.g., brisk walking)																			
Studies where Benefits were found on at least half the EF measures										Studies where Benefits were NOT found at all or on less than half of EF measures									
EF Benefits?	Study	Compared to AC or NT?	Duration in wks	Dose in Minutes [AE portion in brackets]	Fre-quency per Wk	# of Sub-jects	Age Range in Yrs	Mean Age in Yrs^1^	Was a demanding EF measure used?	EF Benefits?	Study	Compared to AC or NT?	Duration in Wks	Dose in Minutes [AE portion in brackets]	Fre-quency per Wk	# of Sub-jects	Age Range in Yrs	Mean Age in Yrs^1^	Was a demanding EF measure used?

Suggestive	Albinet et al., 2010	AC	12	60 [40]	3	12	65–78	71	no	0	Blumenthal et al., 1989	NT	16	60 [45]	3	34	60–83	67	no
Suggestive	Dustman et al., 1984^2^	AC	16	60 [n/a]	3	14	55–70	60	YES	0	Erickson et al., 2011, Leckie et al., 2014, McAuley et al., 2011	AC	52	40 [10–40]	3	65	55–80	67	YES
Clear	Kramer et al., 1999	AC	24	? [n/a]	?	62	60–75	67	YES	0	Fabre et al., 2002	AC	8	60 [45]	2	8	60–76	66	no
Clear	Moul et al., 1995^2^	AC	16	30–40 [30–40]	5	10	65–72	69	no	<50%	Fisher et al., 2011^2^	NT	10	60 [60]	2	32	5–7	6	YES
Suggestive	Predovan et al., 2012	NT	13	60 [15–40]	3	25	57–80	68	YES	0	Legault et al., 2011	AC	17	60 [40]	2	18	70–85	76	YES
Suggestive	Tuckman and Hinkle, 1986	NT	12	30 [30]	3	77	8–12	10	no	0	Mortimer et al., 2012^2^	NT	40	50 [30]	3	30	60–79	68	no
										0	Oken et al., 2006	NT	26	90 [60]	1	45	65–85	72	no
										0	Schmidt et al., 2015	AC	6	45 [45]	2	60	10–12	11	YES
										0	Smiley-Oyen et al., 2008	AC	40	45–50 [25–30]	3	28	65–79	70	no
										0	Voelcker-Rehage et al., 2011	AC	52	60 [35–50]	3	15	63–79	70	no

Means			16	49 [33]	3	33		58 [67]			Means		27	57 [42]	2	34		57 [70]	

Aerobic Exercise Enriched with Cognitive and/or Motor Skill Demands																			
Studies where Benefits were found on at least half the EF measures										Studies where Benefits were NOT found at all or on less than half of EF measures									
EF Benefits?	Study	Compared to AC or NT?	Duration in wks	Dose in Minutes [AE portion in brackets]	Fre-quency per Wk	# of Sub-jects	Age Range in Yrs	Mean Age in Yrs^1^	Was a demanding EF measure used?	EF Benefits?	Study	Compared to AC or NT?	Duration in Wks	Dose in Minutes [AE portion in brackets]	Fre-quency per Wk	# of Sub-jects	Age Range in Yrs	Mean Age in Yrs^1^	Was a demanding EF measure used?
Clear	Chang et al., 2014	NT	8	90 [40]	2	15	5–10	8.5	no	<50%	Chaddock-Heyman et al., 2013, Hillman et al., 2014 & Kamijo et al., 2011	NT	36	120 [77]	5	14	8–9	9	no
Suggestive	Chuang et al., 2015	AC	13	30 [30]	3	8	65–75	68	no	0	Dalziell et al., 2015	NT	16	60 [n/a]	2	23	9–10	10	no
Suggestive	Gallotta et al., 2015^2,3^	AC	20	60 [30]	2	52	8–11	9.5	no	<50%	Davis et al., 2007, Davis et al., 2011	NT	13	40 [35]	5	44	7–11	9	YES
Suggestive	Kim et al., 2011^2^	NT	26	60 [45]	2	26	60–78	68	YES	0	Klusmann et al., 2010	NT	24	90 [30]	3	91	70–93	74	no
Suggestive	Maillot et al., 2012	NT	12	60 [60]	2	16	65–78	74	YES	<50%	Krafft et al., 2014a,b	AC	32	40 [40]	7	22	8–11	9.8	YES
Suggestive	Moreau et al., 2015^2^	AC	8	60 [40]	3	22	18–52	30	no	0	Legault et al., 2011	AC	17	60 [40]	2	18	70–85	76	YES
Suggestive	Staiano et al., 2012	NT	10	30 [30]	1	18	15–19	16.5	index or latent	0	Marmeleira et al., 2009	NT	12	60 [60]	3	16	60–82	68	no
Clear	Williams and Lord 1997	NT	42	50–55 [35]	2	94	>60	72	YES	<50%	Pesce et al., 2013	NT	26	60 [60]	1	83	5–10	7	YES
										<50%	Schmidt et al., 2015	AC	6	45 [45]	2	57	10–12	11	YES

Means^4^			17	55 [40]	2	28		48			Means^5^		23	64 [48]	3	39		36	

AE = aerobic exercise.

A demanding measure = a measure such as the Wisconsin Card Sort Test or Tower of London, on which group differences are often more easily found than on easier EF tasks.

Clear = more EF improvement *and* better EF post-test performance than control group on >67% of measures.

Suggestive = more EF improvement *or* better EF post-test performance than control group on ≥50% of measures.

Index or Latent = creating a composite index from multiple EF measures or looking at the latent variable underlying performance on multiple EF measures is noted because those are likely to be more reliable and tend to be more sensitive than individual EF measures.

^1^The number in brackets includes only studies where the mean age was ≥60 years.

^2^These studies did not include a correction for multiple comparisons. It is unclear which of their results would remain significant had they done that.

^3^Gallotta et al. (2015) randomized by school but appear to have analyzed the data as if they randomized by individual children.

^4^If the FITKids studies are counted as three separate, independent studies, then Kamijo et al. (2011) would have been in the left-hand column as showing suggestive results. The means for enriched aerobic exercise studies where benefits were found on at least half the measures would then be: 19 weeks in duration, 63-min sessions (of which 42 min was aerobic), 2 times per week, 30 subjects per group, and 40 years for age of participants. (Age range and mean age of participants 60 or older would not change).

^5^If the FITKids studies are counted as three separate, independent studies, then Chaddock-Heyman et al. (2013) would have been in the right-hand column as showing no EF benefits and Hillman et al. (2014) would have been in the right-hand column as showing EF benefits on <50% of their measures. The means for enriched aerobic exercise studies where few if any benefits were seen would then be: 22 weeks in duration, 70 min sessions (of which 47 min was aerobic), 4 times per week, 48 subjects per group, and 28 years for age of participants. (Age range and mean age of participants 60 or older would not change).

We predict that EFs should improve most when people are engaged in activities they care deeply about (such as a sport) and for which improving EFs improves performance. Few of the scores of attempts to improve EFs have looked at participants engaged in anything they really care about, yet people learn something best when they need it for something they really care about doing (e.g., Cordova and Lepper, 1996; Olson, 1964). Training de-contextualized skills, isolated from their use in a real-world activity, is unlikely to engender deep personal commitment. There is also evidence that people tend to be far more invested in an activity if they are working together with others toward an important shared goal (as in many sports; Michael et al., 2016).

“Personal characteristics of those leading a program probably have a major impact on how beneficial a program is. That has received too little attention in the EF-training literature and deserves more study. A supportive mentor, who believes in the program and the ability of participants to succeed, who helps build the self-confidence and self-esteem of participants, can be absolutely critical to a program’s success” (Diamond and Ling, in press). This has been demonstrated in many contexts (e.g. Frank, 1961; Hernández et al., 2017; Martin et al., 2000; Rezania and Gurney, 2014).

There has also been little study of the benefits of being outside in nature for EFs (which might characterize brisk walking interventions more than other aerobic exercise interventions that have been investigated). Some intriguing findings about the benefits of nature are emerging, for example: One study found that children with ADHD concentrated better after walking in a park (Faber-Taylor and Kuo, 2009). Indeed, effect sizes were so impressive that the authors suggested that "doses of nature" might serve as a safe and inexpensive way to manage ADHD symptoms. Another study found that a walk in a nature reserve improved performance on a test of attention (Hartig et al., 1991). Yet another study found greater psychological and health benefits from physical activity done outside in nature than from the same activities done inside (Calogiuri et al., 2015). This area of inquiry is worth further investigation.

“The most beneficial programs work, we suspect, because they not only train and challenge EF skills, but they also bring joy, pride, and self-confidence, engender a deep commitment, and provide a sense of social belonging and camaraderie (e.g., team membership). For a similar perspective, see Pesce (2012)” (Diamond and Ling, in press).

It is important to note, however, that a sports program can be destructive if it tears down individuals’ self-esteem, is overly competitive emphasizing being better than someone else rather than better than one’s own past best, abdicates the character-building aspects of the activity, or forgets that first and foremost the activity should be fun (be a source of joy to all who participate). We agree with Vazou (personal communication, Dec. 12, 2017, quoted verbatim with permission) that “sports are not only about the physical demands but also about the motivational climate that might, on the one hand, promote positive peer interactions and make participants feel they belong to the group and are emotionally supported or, on the other hand, undermine feelings of support and positive affect due to competitiveness and rivalry among teammates (when the climate is all about winning and superior ability).”

These points are developed in greater detail in Diamond and Ling (in press). We cannot be sure that other physical activity programs (like real-world sports) will improve EFs. That is only a hypothesis. Few studies have looked at EF benefits from participating in a sport such as tennis or soccer; more investigation of this is needed. On the other hand, many studies have looked at whether aerobic exercise or resistance training benefit EFs; 56% of the former and 75% of the latter have failed to find even suggestive evidence of EF benefits. We can thus state with considerable confidence that aerobic exercise and resistance training approaches that have been studied thus far have generally not succeeded in producing more EF benefit than comparison conditions.

Aside from investigating the EF benefits of aerobic exercise done in the course of engaging in a sport, the intriguing findings that mindful movement activities (such as t’ai chi, taekwondo, Chinese mind-body practices, and Quadrato motor training) consistently improve EFs deserves more study. Also exploring why studies of yoga (another mindful movement activity) have not consistently found EF gains would be of interest.

## 13 Setting the record straight: correcting mischaracterizations of us by Hillman et al. (2018)

### 13.1 What Diamond and Ling (2016) was about

Hillman et al. stated that they “read with great interest Diamond and Ling's (2016) review of the effects of ‘mindful’ and ‘mindless’ physical activity on executive control.” (p. …) That is not what our paper was about. As explicitly stated, (a) Diamond and Ling (2016) was but a short summary of a large systematic review reported elsewhere (in Diamond and Ling, in press) and (b) that in-press review, and its summary in Diamond and Ling (2016), looked at many different activities “including diverse types of computerized cognitive training (especially working memory training), diverse physical activities (such as aerobic exercise, resistance training, coordinative exercise, yoga, and martial arts) as well as other things such as certain school curricula (including Montessori, Tools of the Mind, Chicago School Readiness Program, and PATHS)” (Diamond and Ling, 2016, p. 34). Physical activity was just one of many types of interventions examined.

### 13.2 Criticism that we misunderstood the type of intervention used in the Hillman Lab’s FITKids program

When discussing the Hillman et al. (2014) study, Diamond and Ling (2016, p. 40) correctly noted that “the FITKids Intervention included training in motor skills in addition to aerobic activity.” When discussing the Kamijo et al. (2011) study, Diamond and Lee (2011, p. 960) correctly noted that the FITKids program involved “aerobic activities for 70 min, then motor skill development.” However, Hillman et al. (2018) are correct that in Diamond and Ling (2016) the FITKids study by Kamijo et al. erroneously appears under plain aerobic exercise. We corrected that in page proofs, but that correction did not make it into the printed version. We acknowledge the error and sincerely apologize for it. We do not blame Hillman et al. for being displeased about that. That error does not affect our conclusions, however.

Hillman et al. (2018) are incorrect in asserting that Diamond and Ling (2016) used the Kamijo et al. (2011) or the Hillman et al. (2014) studies as “pillars of their argument that ‘mindless’ physical activity does not promote changes in executive function.” We did not use these as pillars; we had no need to use them as pillars; the literature is replete with examples of plain aerobic exercise not benefitting EFs. We used these two studies as examples where benefits were claimed but we were doubtful, and used the Hillman et al. (2014) study as an example of that under more-cognitively-demanding aerobic exercise, not ‘mindless’ physical activity.

#### 13.2.1 Having only a no-contact group as the only control condition in a study

Although we did not criticize Kamijo et al. (2011) or Hillman et al. (2014) for not including an active control group, we did say that studies without an active control condition are weaker, and these studies from Hillman’s lab did not include an active condition. Hillman evidently seemed to feel a need to defend himself, for Hillman et al. (2018) wrote, “Diamond takes issue with the use of non-contact control groups to compare against intervention groups. Although such a perspective that favors a more active control group receiving a benign intervention is meritorious for a number of important reasons, *it should never be assumed that this is the best comparison for all studies*” (emphases added).

Hillman et al. (2018) are wrong in that; it should *always* be assumed that a no-treatment condition alone is insufficient for any and all studies. We could cite scores of reviews and textbooks on this, but it is probably sufficient to cite just two. Here is Green et al. (2014, p. 766): “There is general agreement that active control groups are necessary, as simple test-retest/no contact/passive control groups fail to rule out too many possible confounds to allow results to be meaningfully interpreted.” In their landmark review of brain-training programs, Simons et al. (2016) place studies with only a no-treatment control condition under the category, “**Substantial Problems**. These problems mean that a study can provide only ambiguous or inconclusive evidence for the effectiveness of an intervention. Findings from papers with these problems should be treated as tentative at most. They should not be used in determining public policy” (p. 171) and go on to say, “Passive control group: Studies comparing an intervention group to a waitlist or no-contact control group cannot attribute causal potency to the intervention itself. Any differences between the treatment and control group can account for the difference (e.g., motivation, expectations, engagement, interaction with the experimenter)” (Simons et al., 2016, p. 171).

This is not to say that a no-treatment control group has no value; it is simply insufficient in and of itself. Indeed, Diamond and Ling (2016) made the same points about what a no-treatment group *does* control for as Hillman et al. (2018) made in their criticism of Diamond and Ling. We had written:

“(e) A comparison group had to be included. (To conclude that what individuals did between Times 1 and 2 produced the improvement at Time 2, there needs to be evidence that without that activity there is less improvement at Time 2, even in those who were also tested at both timepoints. Without that there is no way to tell if improvements might have happened anyway from just having taken the assessment measures before (practice effects) or just from normal developmental improvement in the abilities tested].)” (Diamond and Ling, 2016, p. 35)

This does not mean that all choices for an active control condition are good ones. For example, the full benefits of physical activity might not be evident from studies that compare less or more physical activity or two different types of physical activity. It might be better to have an active control group that does something other than physical activity (such as sedentary activities like arts and crafts or reading) in *initial* evaluations of EF benefits from a physical activity intervention.

In addition, Diamond has also criticized studies with *only* an active control condition for not also including a no-treatment condition (Diamond, 2014). When both experimental groups show the same improvement and outcome, without a no-treatment group it is not possible to determine if both conditions produced comparable benefits, or whether neither condition produced a benefit and improvements were simply due to test-retest practice effects or normal developmental processes. Thus, it is difficult to understand why Hillman et al. took issue with what we had to say. Their defense of designs with only a no-treatment control condition appears misplaced.

### 13.3 Criticism that we did not consider dose, duration, intensity, etc

Hillman et al. (2018) bemoaned the absence of a “discussion of mode, intensity, or duration of the interventions” in Diamond and Ling (2016). Perhaps they missed Point 2 under “Conclusions that emerge from the various studies of different methods of trying to improve EFs” that spanned pages 36–37, where we discussed findings within and across studies concerning dose, duration, and other variables. We did not consider intensity, however, as it was not relevant to any type of intervention other than physical activity. In hindsight, we regret that we did not include that.

Hillman et al. (2018) are correct, however, that Diamond and Ling (2016) did not provide values for each of those variables for each study. There was simply no room to include all of that in the brief summary of our large systematic review. Readers are referred to Tables 2 and 3 (which span well over 100 pages) in Diamond and Ling (in press) for detailed information on type of intervention, dose (minutes per session), frequency (days per week), duration (number of weeks), whether the activity was done alone or with others, compliance rate (percentage of sessions attended), attrition rate (percentage of participants who dropped out), characteristics of the active control condition(s), whether there was a no-treatment or business-as-usual condition, whether there was random assignment, and whether testers were blind to group assignment for each study. The volume that Diamond and Ling (in press) will appear in was due out in 2016 and we hope it will be released any day now.

We decided not to report whether an intervention achieved changes in fitness because that was not relevant to the vast majority of studies in our review (e.g., studies of cognitive training, mindfulness, or school curricula) and because improvements in fitness and improvements in cognition (including EFs) have repeatedly been found to be uncorrelated (e.g., *meta-analyses*: Etnier et al., 2006; Young et al., 2015; *review*: Kramer and Erickson, 2007; *also see* Blumenthal et al., 1989; Davis et al., 2011; Smiley-Oyen et al., 2008). We discuss what cognitive ability each outcome measure assessed, whether a measure bore a close similarity to what was done during training, and where appropriate, the difficulty of an outcome measure, but we did not rate the “quality” of cognitive assessments because (although cognition is our specialty) we know of no truly accurate way to rate the quality of the measures.

### 13.4 Spurious attacks on our conclusion that school programs and martial arts have generally produced better ef outcomes than aerobic-exercise or resistance-training interventions

Hillman et al. (2018) found unusual grounds on which to take issue with our conclusion about greater EFs benefits from school curricula and a martial arts program.

They asserted that *Tools of Mind* (a curriculum for preschool and kindergarten) was “our program,” which would have meant that when Diamond et al. (2007) evaluated *Tools of Mind,* we were evaluating our own program or one we had a hand in developing. That claim has no merit. *Tools of Mind* was independently developed by Bodrova and Leong (1996). It was developed by one group and independently evaluated by a completely separate, unrelated group, as should be done for evaluating any program. (It is not clear if that was true of the FITKids program evaluated by Hillman’s lab. FITKids appears to have been developed at the University of Illinois, where Hillman’s lab has been located. Nowhere is the independence of the program and those evaluating it made clear.)

Hillman et al. (2018) further questioned the findings for *Tools of Mind,* putting forward the odd claim that randomization at a group level makes it more difficult to draw causal inferences than randomization at the individual level. There is no basis for that claim (as long as each design has sufficient power). They seem to base their claim on reasoning that if a different person administers the experimental and control conditions, different outcomes might be due to personal characteristics of the individual administering the condition rather than to properties intrinsic to either condition.

First, that applies regardless of the level at which randomization occurred. Second, typically cluster-randomized designs have multiple individuals administering each condition, thus diminishing the likelihood that systematic differences between those administering one or the other condition account for observed differences in group outcomes. Third, group differences can be found because those administering the conditions expected one condition to be more beneficial; that can occur if the same person administered both conditions or different people did.

Hillman et al. (2018) falsely claimed that Diamond and Ling (2016) reported that *Tools of Mind* was more successful than other approaches. Diamond and Ling (2016) reported that school programs in general had been more successful in improving EFs than other approaches, and *Tools of Mind* is a school program, but Diamond and Ling claimed no greater success for *Tools of Mind* than for other school programs.

Finally, Hillman et al. (2018) wrote, “Diamond’s assumptions concerning Tae-Kwon Do versus regular physical education (Lakes and Hoyt, 2004) lack merit, as physical education has been routinely demonstrated to be both cognitively engaging and demanding given the requirement to plan and learn complex motor skills, game/competition strategy and rules, regulate physical behaviors, and social interaction.” We respectfully disagree. Physical education *should* be as Hillman et al. describe, but too rarely is. That is why it is so often used as the control condition in studies of physical activity interventions. Moreover, the mindfulness aspects of traditional taekwondo have never been part of standard physical education. Taekwondo simply as a physical activity, which is counter to the taekwondo tradition and which Trulson (1986) found produced negative outcomes rather than benefits, would be more similar to standard physical education.

In closing, although the results for aerobic-exercise or resistance-training interventions improving EFs have thus far been discouraging, we predict that physical activity programs (or arts programs or social service programs, etc.) that challenge EFs in varied ways and engage children’s hearts and souls, helping them feel proud, self-confident and supported by people who are there for them and believe in them will indeed improve EFs in major and significant ways. The way for us to find the best approaches for improving, or restoring, EFs is for us to work together, not attack one another.

## Footnotes

1 Whereas Davis and Krafft made very clear that their publications were reporting on the same research study, the papers from Hillman’s lab on the FITKids program (Chaddock-Heyman et al., Hillman et al., Kamijo et al., and Monti et al.) did not. Only in the Donnelly et al. review is it mentioned that Hillman et al. included participants already included in the earlier reports. In addition, two of those papers report the results for only one outcome measure (Kamijo et al. reported results only for the Sternberg task; Monti et al. reported results only for relational memory). Were the outcome measures all administered at the same time pre- and post-intervention and then individually reported in separate research reports?. In their seminal review of brain training studies, Simons et al. (2016) raise a red flag about such practices: “[I]f other papers by the same team report different outcome measures from what appears to be the same intervention, the results and conclusions of each paper must be viewed with skepticism. Such a pattern of publication implies that other outcome measures—those that did not ‘work’—might never be reported, thereby muddying the interpretation of all of the published results.” (p. 119)

2 Diamond and Ling (2016), p. 42: “Studies are also sorely needed that systematically vary dose, frequency, and duration…[and] studies are needed that vary other characteristics of the activities.”

3 Diamond and Ling (2016), p. 40: “Most sports place demands on each of the EFs. Participants need to remember complex movement sequences, mentally work with lots of information, processing in real-time cues such as people’s positions and where they will likely go next (for ball sports, cues about the ball’s location and trajectory), mentally compare the present situation with past ones, and use that to predict what is likely to happen next or down the line (i.e., they must use WM). Participants need to inhibit attending to distractions and keep their attention focused; they must inhibit a planned action when that is suddenly no longer a good idea and inhibit what might be their first inclination, such as the temptation to try to score oneself rather than passing (i.e., they must use inhibitory control). And, they must use cognitive flexibility: The situation is constantly changing. Participants must quickly and accurately evaluate and respond to those changes, flexibly switching plans in real time, adjusting to the unexpected, adapting to complex and rapidly changing conditions. The situation they are faced with at any moment is often different from anything they have faced before. They can never know for sure what someone else will do; at best they can only predict. Some of this can become automatized and no longer require top-down control, but (a) that is less true for people relatively new to a sport and (b) typically the difficulty of what one is facing keeps increasing. As other players or opponents get better at the sport, the inherent difficulty of what one is faced with increases, providing constant challenge.”
